# Splenic and Portal Vein Thrombosis after Splenectomy: A Case Report

**DOI:** 10.5334/jbsr.2947

**Published:** 2022-12-08

**Authors:** Thomas Saliba, Hanna Salame, Denis Tack

**Affiliations:** 1 ULB, BE; 2 Epicura Hospital, BE

**Keywords:** portal vein thrombosis, splenectomy, splenic vein thrombosis, thrombosis, post-operative thrombosis

## Abstract

Portal and splenic vein thrombosis are uncommon, potentially fatal post-operative complications following splenectomy. These thrombotic events may be asymptomatic or present with non-specific symptoms. Therefore, imaging is important for diagnosis. The risk of thrombosis is linked to spleen size, pre-operative thrombocytopenia and surgical technique. We present the case of a 40-year-old man who underwent curative and diagnostic laparotomic splenectomy following chronic thrombocytopenia and concurrent splenomegaly who subsequently developed extensive portal and splenic vein thrombosis.

**Teaching Point:** Portal and splenic vein thrombosis after splenectomy is a relatively uncommon but important diagnosis in which radiology has a pivotal role.

## Introduction

Portal and splenic vein thrombosis are potentially fatal complications following splenectomy, with a study-dependent incidence ranging from 0.8% to 55%, and with a more recent study from 2018 reporting a rate around 8% [1,2,3]. This complication can be a challenging clinical diagnosis, asymptomatic in around 95% of cases or with non-specific symptoms like fever, pain, nausea, anorexia, vomiting or diarrhoea [3,4,5]. Nevertheless, undiagnosed, this pathology is fatal in up to 5% of cases [2].

## Case History

A 40-year-old man presented with thrombopenia, found during a routine pre-operative workup for an unrelated procedure, and splenomegaly. The patient was known to have thrombopenia for 10 years. A previous workup for thrombocytopenic purpura was performed, proving negative as immunoglobulin therapy was unresponsive. An FDG-PET-CT only showed hypermetabolic splenomegaly. A splenectomy with diagnostic and therapeutic intention was suggested to exclude low-grade lymphoma, but no action was taken at the time. At the current presentation, an open splenectomy was performed. There was no perioperative complication. The enlarged spleen had a weight of 1.89 kg. Postoperatively, the patient presented with a 10-day persisting fever despite antibiotics; he was referred for a computed tomography (CT) exam to exclude late postoperative complications or infection. The CT exam, performed 15 days postoperatively, revealed splenic vein thrombosis, extending into the intrahepatic segment of the left branch of the portal vein (Figures 1a and 1b–c). A PET-CT was performed four days later searching for infection, confirming a non-hypermetabolic, and most likely non-tumoral, thrombus (Figure 2). The patient was subsequently treated and discharged.

**Figure 1a:**
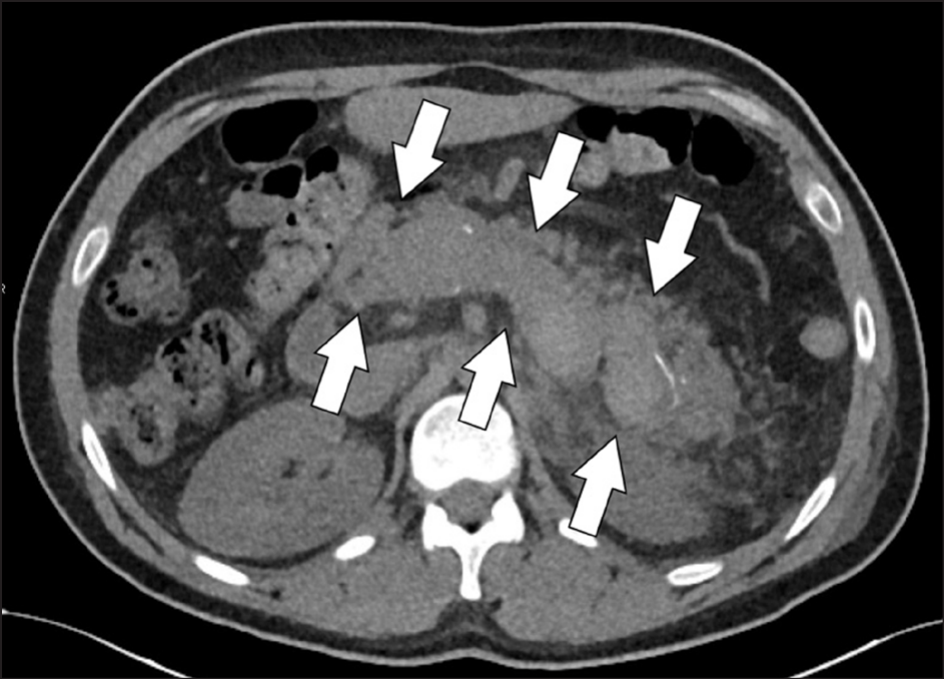
Axial unenhanced CT-slice revealing a spontaneously hyperdense splenic vein thrombus.

**Figure 1b and c:**
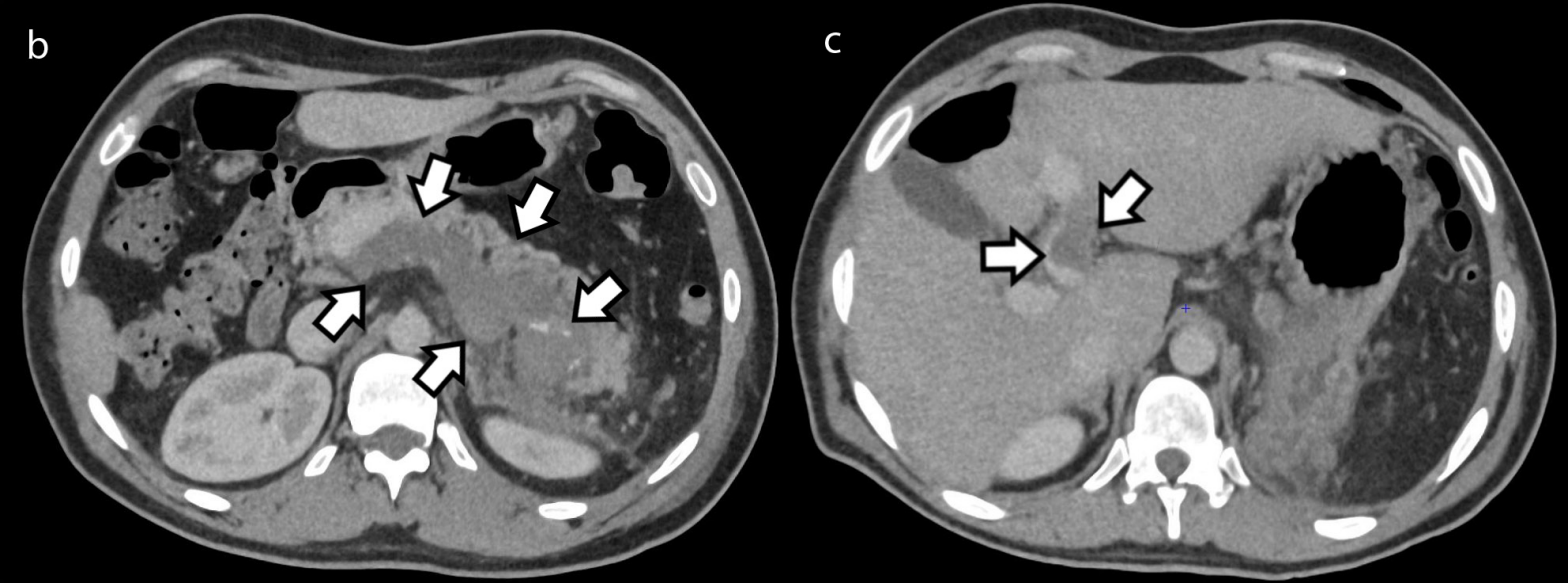
Axial contrast-enhanced portal phase CT slices at the level of the splenic vein (Figure 1b) and portal vein (Figure c) confirming a thrombosis of the splenic vein, extending into the portal vein as well as the part of the intrahepatic segment of the left branch of the portal vein (arrows).

**Figure 2:**
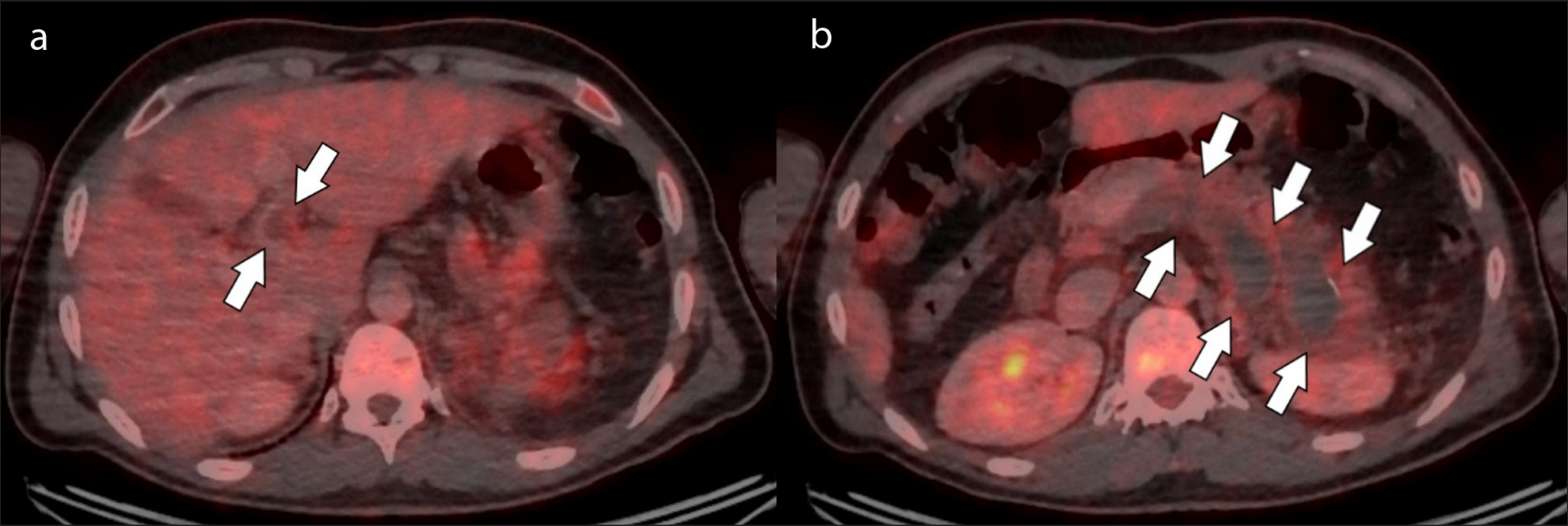
Axial FDG-PET/CT slices at the level of the splenic vein (Figure 2a) and portal vein (Figure 2b) showing absence of metabolism within the thrombus and some hypermetabolic infiltration of surrounding fat (arrows).

## Discussion

As most cases are clinically asymptomatic or non-specific, radiology is crucial in diagnosing splenic and portal vein thrombosis. Contrast-enhanced CT exam is superior to ultrasonography [1,5]. Preoperative radiological criteria predictive of thrombosis include splenic vein diameter. Wider splenic veins (>12.3 mm (95% CI 10.5–14.1) seem to be more susceptible to thrombosis compared to veins with a normal diameter of 9.02 mm (95% CI 8.5–9.5 on average) [1]. In addition, pre-operative splenomegaly has been linked with venous thrombosis [1]. In a study by Péré et al., univariate analyses of their patients showed that a pre-operative splenic vein diameter >10 mm generated an odds ratio of 4.92 in favour of thrombosis, as did an estimated splenic weight >500 g, with an odds ratio of 8.72. Other studies however reported different cut-off points [1,2,6]. Pre-operative thrombocytopenia was associated with thrombosis with an odds ratio of 2.17 [1]. Some studies identified the surgical procedure as a risk factor, with laparoscopic surgery being associated with increased thrombosis risk compared to laparotomy, whereas others found no relationship between these surgical techniques [1,6].

Portal or splenic vein thrombosis treatment is controversial. In some centres, treatment consists of intravenous heparin or low-molecular weight heparin (LMWH) followed by oral anticoagulants for 3–6 months, while others recommend simultaneous LMWH and warfarin followed by warfarin for 3–6 months [2,5]. In acute thrombosis, thrombolysis could be attempted, especially if it is extensive or involves the superior mesenteric vein [2]. Importantly, treatment efficacy and prognosis are time-critical [1].

Prophylaxis is also the subject of discussion, post-operative anticoagulation being standard in some, but not all, institutions [1,5].

## Conclusion

Portal and splenic vein thrombosis are uncommon and often asymptomatic post-splenectomy complications, and contrast-enhanced CT exam is the preferred imaging modality. If the patient presents with pre-operative risk factors for thrombosis, including a splenic vein diameter >10 mm or a spleen weight estimated >500 g, a follow-up exam to diagnose and treat this severe condition could be taken into consideration [1].
